# Decrease of the plasmatic endocan cleavage ratio is associated with the hyperinflammatory phenotype of acute respiratory distress syndrome

**DOI:** 10.1186/s13054-019-2537-z

**Published:** 2019-07-11

**Authors:** Alexandre Gaudet, Erika Parmentier, Nathalie De Freitas Caires, Lucie Portier, Sylvain Dubucquoi, Julien Poissy, Thibault Duburcq, Maxence Hureau, Philippe Lassalle, Daniel Mathieu

**Affiliations:** 1 Center for Infection and Immunity of Lille, University of Lille, U1019, UMR 8204, CIIL, F-59000 Lille, France; 2 CNRS, UMR 8204, F-59000 Lille, France; 3 INSERM, U1019, F-59000 Lille, France; 4 CHU Lille, Pôle de Réanimation, Hôpital Roger Salengro, F-59000 Lille, France; 5 Lunginnov, 1 rue du Pr Calmette, F-59000 Lille, France; 6 CHU Lille, Institut d’Immunologie, Centre de Biologie Pathologie Génétique, F-59000 Lille, France

Dear editor,

Several studies have reported the identification of pro-inflammatory subphenotypes of acute respiratory distress syndrome (ARDS) which seem to be the most likely to respond to targeted treatments, such as restrictive vascular filling, higher PEEP, or statins [1]. In these studies, vasopressor use, low platelets, and low bicarbonate appeared as routine markers of hyperinflammatory ARDS [1]. Endocan and its major catabolite p14 have been reported as novel biomarkers in ARDS [2], yet their potential utility in the characterization of ARDS phenotypes has never been explored.

We hereby present the results of a post hoc analysis based on the data from a previously published prospective cohort of severe septic patients [3]. Patients with a diagnosis of ARDS within 72 h following enrollment were included in this analysis. We considered ARDS as belonging to the HIP group when the 3 following criteria were present at the time of diagnosis: vasopressor use, platelets < 150 G/L, and bicarbonate < 22 mmol/L [1]. Measurements of endocan and p14 were performed at the time of diagnosis of ARDS and 24 h later if biological samples were available. Plasmatic endocan cleavage ratio (ECR) was calculated as previously described [4]. The aim of this analysis was to assess if static measurements of blood endocan and ECR at time of diagnosis of ARDS, as well as their variations within 24 h, were different between the HIP and NHIP subgroups of ARDS.

Thirty-nine patients with a diagnosis of ARDS were included in this analysis. Plasmatic levels of endocan and ECR were measured at the time of ARDS in every patient and were repeated 24 h later in 29 patients. Patients’ baseline characteristics are described in Additional file 1. ECR variation over 24 h was the only parameter to be found significantly different in the HIP group (− 7% [− 19%; − 5%]) by comparison with the NHIP group (6% [− 3%; 16%]) (*p* < 10^− 2^) (Fig. 1) and to show clinically relevant diagnostic values according to ROC analysis (AUC = 0.84 (95% CI 0.69–0.94; *p* < 10^− 2^)) (Fig. 2). A variation of ECR < − 4.5% was found as the best cutoff for the diagnosis of HIP ARDS according to the Youden index, with a sensitivity at 0.86 and a specificity at 0.82.

**Fig. 1:**
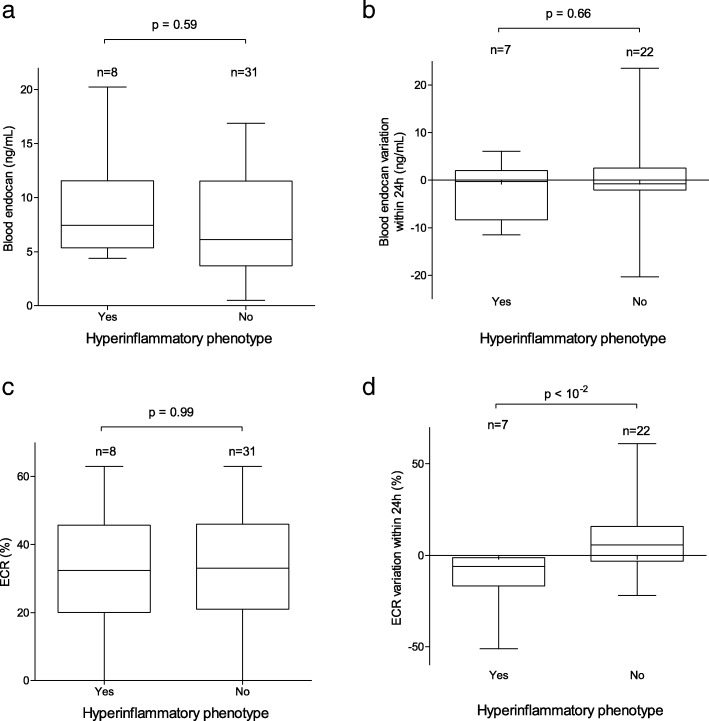
Endocan and ECR plasmatic levels at time of ARDS and variations over 24 h in HIP and NHIP ARDS. We found no difference between HIP and NHIP groups for blood concentrations of endocan (**a**), variations of endocan within 24 h (**b**), and ECR (**c**). ECR variation over 24 h in the HIP group was significantly different to that of the NHIP group (**d**). Box plot shows the median (horizontal line) and IQR (25th–75th percentile) (box). The whiskers show the lowest data within 1.5 IQR of the lower quartile and highest data within 1.5 IQR of the upper quartile. We used the Mann-Whitney test for comparisons of two groups of continuous variables and the chi-square test for comparisons of categorical variables. ARDS: acute respiratory distress syndrome; ECR: endocan cleavage ratio; HIP: hyperinflammatory phenotype; NHIP: non-hyperinflammatory phenotype

**Fig. 2:**
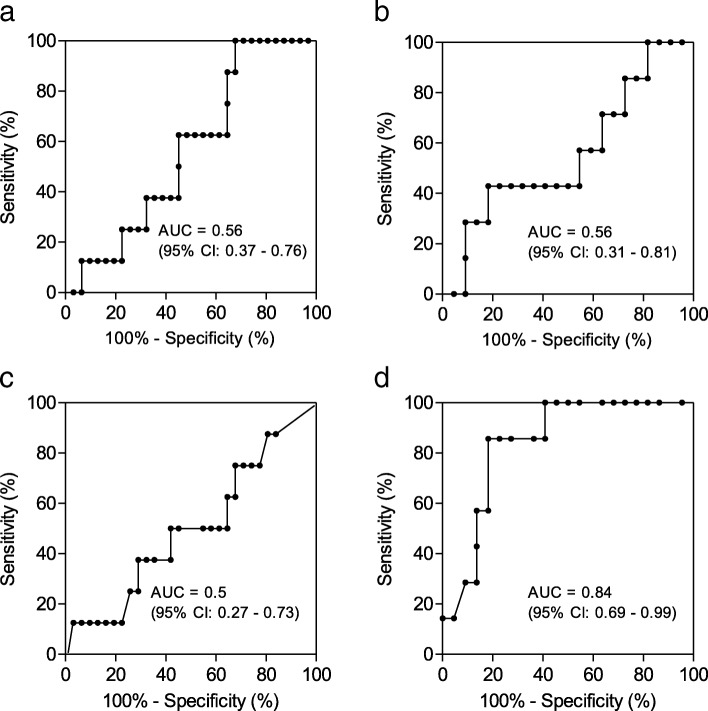
ROC analysis of endocan plasmatic levels at time of ARDS (**a**) and their variations over 24 h (**b**), as well as ECR plasmatic levels a time of ARDS (**c**) and their variations over 24 h (**d**) for the diagnosis of hyperinflammatory subphenotypes of ARDS. ARDS: acute respiratory distress syndrome; ECR: endocan cleavage ratio; HIP: hyperinflammatory phenotype; NHIP: non-hyperinflammatory phenotype

These results suggest that the variation of ECR 24 h after the diagnosis of ARDS seems to be a discriminant biomarker to identify hyperinflammatory subphenotypes of ARDS. New studies are warranted to comfort these preliminary observations.

## Additional file

Additional file 1: Cohort baseline characteristics. Continuous and categorical variables are described as median [interquartile range] and number (percentage), respectively. *COPD* chronic obstructive pulmonary disease *SOFA* Sequential Organ Failure Assessment *ICU* intensive care unit *SAPS 2* Simplified Acute Physiology Score 2 *LIPS* Lung Injury Prediction Score. (DOC 45 kb)

## Data Availability

The datasets used and/or analyzed during the current study are available from the corresponding author on reasonable request.

## Abbreviations

ARDS: Acute respiratory distress syndrome
ECR: Endocan cleavage ratio
HIP: Hyperinflammatory phenotype
NHIP: Non-hyperinflammatory phenotype
