# Supplementary material for: Decrease of the plasmatic endocan cleavage ratio is associated with the hyperinflammatory phenotype of acute respiratory distress syndrome
Source: Crit Care. 2019 Jul 11;23:252. doi: 10.1186/s13054-019-2537-z (PMC6625000; doi:10.1186/s13054-019-2537-z)
Supplement: Supplementary file 1 — Cohort baseline characteristics. Continuous and categorical variables are described as median [interquartile range] and number (percentage), respectively. COPD chronic obstructive pulmonary disease SOFA Sequential Organ Failure Assessment ICU intensive care unit SAPS 2 Simplified Acute Physiology Score 2 LIPS Lung Injury Prediction Score. (DOC 45 kb) [file 13054_2019_2537_MOESM1_ESM.doc]

**Additional File 1. Cohort baseline** characteristics

| **Variables** | **All patients**  **(n = 39)** |
| --- | --- |
|
| Age (years) | 59 [44 – 68] |
| Sex (male) | 28 (72%) |
| Chronic comorbidities  COPD  Smoker  Cardiomyopathy  Chronic kidney failure  Cirrhosis | 4 (10%)  12 (31%)  12 (31%)  1 (3%)  7 (18%) |
| Sepsis severity on enrolment  Severe sepsis  Septic shock | 8 (21%)  31 (79%) |
| Site of infection on enrolment  Soft tissues  Respiratory  Urinary  Digestive  Other | 12 (31%)  20 (51%)  1 (3%)  3 (8%)  3 (8%) |
| Biomarkers on enrolment  CRP (mg/L)  PCT (ng/mL) | 227 [97 – 273]  22.4 [4.1 – 57.4] |
| Prognostic scores on enrolment  SAPS 2  SOFA | 65 [50 – 75]  11 [8 – 13] |
| Organ SOFA on enrolment  Pulmonary  Renal  Hepatic  Circulatory  Neurological  Haematological | 2 [2 – 3]  0 [1 – 3]  0 [0 – 2]  4 [3 – 4]  4 [0 – 4]  0 [0 – 1] |
| Mortality  Day 28  ICU discharge | 13 (33%)  12 (31%) |
| ICU length of stay (days) | 15 [7 – 24] |
| Mechanical ventilation on enrolment | 29 (74%) |
